# Assessment of the Thermal Properties of Gypsum Plaster with Plastic Waste Aggregates

**DOI:** 10.3390/ma17071663

**Published:** 2024-04-04

**Authors:** Alejandra Vidales-Barriguete, Eva Barreira, Susana Gomes Dias

**Affiliations:** 1 Departamento de Tecnología de la Edificación, Escuela Técnica Superior de Edificación, Universidad Politécnica de Madrid, Avenida Juan de Herrera, 6, 28040 Madrid, Spain; alejandra.vidales@upm.es; 2 CONSTRUCT-LFC, Civil Engineering Department, Faculty of Engineering (FEUP), University of Porto, 4200-465 Porto, Portugal; up201905836@g.uporto.pt

**Keywords:** plastic waste, gypsum composites, recycling, sustainable construction, thermal properties

## Abstract

Building material manufacturers must support new production models that encourage the manufacture of more efficient and sustainable products. This includes thinking about savings in the use of raw materials, a contribution to the energy efficiency of buildings during their useful life, and a reduction in the generation and deposit of waste in landfills. In this research, an analysis of the thermal properties of gypsum composites added with plastic waste is carried out using the most common methods, the steady state method and the transient plane source method, and the effect of water saturation on these composites is tested. The results show an improvement in the thermal performance of the composites (values reduced with respect to the reference by 4–7%), despite their heterogeneity, as well as a variation in the measurements carried out, depending on the method used for the measurements (variation up to 10%). It is also found that the degree of humidity negatively affects the thermal conductivity coefficient but, on the contrary, this coefficient is not altered in the composites with plastic waste, due to their lower hygroscopicity. Therefore, it is considered that the proposed eco-plasters are a good alternative to traditional plasters, with which to contribute to the achievement of the objectives of the current European directives on waste and circular economy.

## 1. Introduction

Nowadays, construction materials for residential buildings have to meet increasingly demanding requirements not only in terms of minimizing the use of natural resources during their manufacture, but also in terms of the energy efficiency of the material already in place and the reduction in waste generation after its useful life. In this sense, many European directives are being enunciated to try to achieve these standards.

Directive (EU) 2018/851 amending Directive (EU) 2008/98/EC on waste establishes a circular economy package for Member States to support sustainable production and consumption patterns by encouraging the design, manufacture, and use of efficient products [1,2]. This is why innovation in building materials is essential for researchers, manufacturers, designers, and contractors.

Gypsum is a traditional material with large quarries in Europe (Spain, Germany, Italy, and France), used mainly as an interior finish for rooms in houses, either as trim and plaster or in the form of panels, due to its excellent hygrothermal properties and ease of installation [3]. On the other hand, polymers are also materials that are widely used on site due to their great versatility. They are durable, economical, lightweight, electrical insulators, anticorrosive, etc., but this fact, in turn, hinders their recycling methods and poor management of polymeric waste implies harmful environmental impacts [4,5].

One of the solutions to the problem of plastic waste is to incorporate it into the matrix of one of the so-called “traditional building materials” (gypsum, cement, or ceramics), in order to give it a new life and optimize some of its properties, such as, for example, an improvement in energy efficiency. The characteristics of materials influence the energy consumption of buildings, since they are part of the passive efficiency of buildings, which should be considered as one of the measures of their energy performance [6]. The value of the thermal conductivity of the thermal envelope of a building, of which the interior finish of each room (plaster or plasterboard) is a component, is one of the indicators that are considered to evaluate and compare the energy efficiency in European buildings, together with the energy consumption in installations such as heating and domestic hot water, and with which the parameter for obtaining the Energy Rating is formed [7].

Two methods are commonly used to measure the thermal conductivity of building materials, the steady state method and the transient plane source method. The former measures thermal conductivity by establishing a temperature difference that does not change with time, while the latter focuses on measuring the time-dependent energy dissipation process of a specimen [8].

Some researchers have managed to improve the energy efficiency of building materials through polymeric waste. This is the case of Abdel Tawab, who introduces recycled plastic bags for the manufacture of concrete blocks, achieving thermal conductivity coefficients up to 16% lower than the reference blocks, measured using the transient technique with an apparatus designed in his Bioengineering Laboratory [8]. Kumi-Larbi, who uses single-use LDPE drinking water sachets (widely used in Africa) melted and mixed with sand to form blocks with very good thermal behaviour, measured through a non-destructive test based on the transient plane source technique using a Hot Disk M1 analyser model from the commercial house Hot Disk (Göteborg, Sweden) [9]. Wesam manufactures blocks with PET and PU plastic bottle waste, achieving thermal conductivity values between 0.15 and 0.30 W/mK, by means of transient plane source [10]. Wang introduces high-impact recycled polystyrene (HIPS) as a substitute for sand in the cement mortar, achieving a reduction in thermal conductivity of up to 87% compared to that of the reference mortar, measured using transient plane source equipment [11]. Sosoi manufactures concrete in which he replaces part of the sand with PET waste and polystyrene granules, achieving a lighter concrete with a thermal conductivity value 25% lower than the reference sample, obtained using transient plane source equipment [12]. Senhadji studied the possibilities of adding polyvinyl chloride (PVC) waste as an alternative fine aggregate in ecological mortars, achieving reductions of up to 41% in thermal conductivity with respect to a traditional mortar, whose measurements were obtained through a Quick Thermal Conductivity Meter (QTM 30) device using the steady state method for 90 days [13]. Artid Poonyakan made mortars incorporating HDPE, LDPE, PP, and PET plastic waste in different percentages and dimensions, achieving a thermal conductivity coefficient 31% lower than other authors, using NETZSCH HFM 436 heat flow equipment [14]. M.R. Ali also produces concretes with thermal conductivity coefficients 30% lower than other similar investigations, incorporating polyethylene beads, measured using the transient plane source method [15]. And Shaik Inayath makes concretes with three types of recycled plastics, with which he obtains a 35–65% reduction in thermal conductivity compared to the reference, measured using AFOX50 heat flow equipment [16].

Other authors have also studied the effect of incorporating plastic waste in gypsum matrices. Herrero who used recycled rubber in three different particle sizes (0–0.6 mm, 0.5–2.5 mm, and 2.5–4.0 mm), obtaining small fluctuations but with all values lower than those of the reference material measured using a “thermal house model” using the steady state method [17]. Pedreño-Rojas fabricated commercial gypsum specimens in which he included polycarbonate residues, obtaining a 42% reduction in the thermal conductivity coefficient, measured by means of the ISOMET-2114 instrument, working according to the transient plane source method [18]. Del Río Merino considered the manufacture of gypsum mortars with EPS and XPS residues, with which he improved the thermal properties by 40%, values were obtained through the C-Therm instrument that uses the transient plane source method [19]. Balti, who introduced expanded polystyrene (EPS) waste from the shredding of unpacking parts and polyester waste from packaging ropes in the industry, achieved a reduction of about 50% for the thermal conductivity coefficient, obtained using a Hot Disk 2500S instrument from the commercial house Hot Disk (Göteborg, Sweden) that operates using the transient plane source method [20]. On the other hand, Oliveira elaborated plaster specimens with cellulose waste and expanded polystyrene, achieving a lower value of up to 48% with respect to the reference plaster, through the Thermal Conductivity Tester DTC-300 instrument that operates with steady-state heat flow meters [21]. Bouzit also developed a gypsum mortar with expanded polystyrene beads, in which the thermal conductivity decreased between 26% and 45% with respect to commercial gypsum, obtained using a Small Hot Box, under steady state conditions [22].

This literature review showed that, in all studies assessing thermal conductivity of gypsum mortars incorporating waste, only a single measurement method was used without double-checking using other methods. For this reason, the aim of this research is to check whether there are significant differences between the values obtained using the two most common methods for measuring thermal conductivity (the steady state method and the transient plane source method) for gypsum products incorporating plastic waste from construction and demolition waste. Another innovative aspect of this study is the evaluation of the effect of the incorporation of these components in the variation of thermal conductivity for higher moisture contents. Analysing the accuracy of these procedures will allow us to more accurately evaluate the energy efficiency of these products and to achieve a more efficient eco-plaster that helps to reduce both the energy consumption of buildings and the amount of plastics deposited in landfills and the use of raw materials (water and aljez stone).

## 2. Methodology

### 2.1. Materials

The samples used in this experimental campaign were prepared in the laboratory, following the requirements of the standard UNE EN 13279-2 [23]. The materials used were gypsum, plastic pellets, and water. The gypsum, manufactured by Placo^®^ Saint-Gobain (Madrid, Spain), had a density of 2.81 g/cm^3^ and the density of the plaster was 2.72 g/cm^3^, both measured using a helium pycnometer [24]. The plastic pellets were prepared from the waste of the recycling industry of electric cables and the supplier was Lyrsa Álava recycling plant. They were a mixture of different thermoset and thermoplastic polymers, with a granulometric size inferior to 3 mm and a density of 1.35 g/cm^3^, obtained using a helium pycnometer. Water was derived from the Canal de Isabel II in Madrid and is in accordance with the standard UNE EN 13279-2 [23]. All the samples were made with dimensions of 303 ± 2.5 mm × 303 ± 1.8 mm × 32 mm and with a water/plaster mass ratio of 0.8.

**Figure 1:**
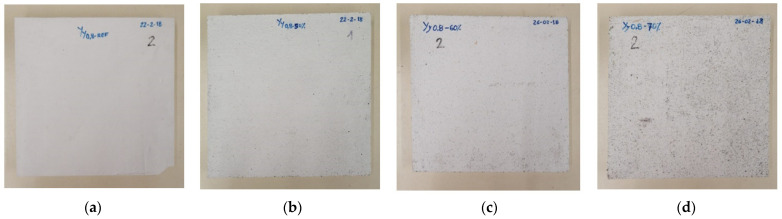
Gypsum specimens under study. (**a**) Reference; (**b**) with plastic waste aggregates with a ratio of 50%; (**c**) with plastic waste aggregates with a ratio of 60%; and (**d**) with plastic waste aggregates with a ratio of 70%.

Four sets of specimens were used, each one with three samples, resulting in a total of twelve samples (Figure 1). A set of classical gypsum plaster was used as reference and, in the other three sets, plastic waste aggregates from cables (PW) were incorporated with a ratio of 50%, 60%, and 70% of the aggregate over the gypsum mass. Figure 1 shows the external visual appearance of the specimens as more plastic waste is incorporated. In the specimen with 50% PW, granulation on the surface is hardly detected; however, in the specimen with 70% PW, the granulation is clearly visible.

The gypsum and the plastic waste were dried and mixed before adding the water, to obviate the waste from floating, to then be mechanically mixed with water. The storage of the samples also complied with the standard UNE EN 13279-2 [23]. The main mechanical and hygrothermal properties of these new materials, as well as their fire behaviour, can be found in previous studies [24,25,26,27].

### 2.2. Equipment

Two different methods were used to measure the thermal conductivity of the samples, (i) the heat flow meter (HFM) and (ii) the modified transient plane source (MTPS).

**Figure 2:**
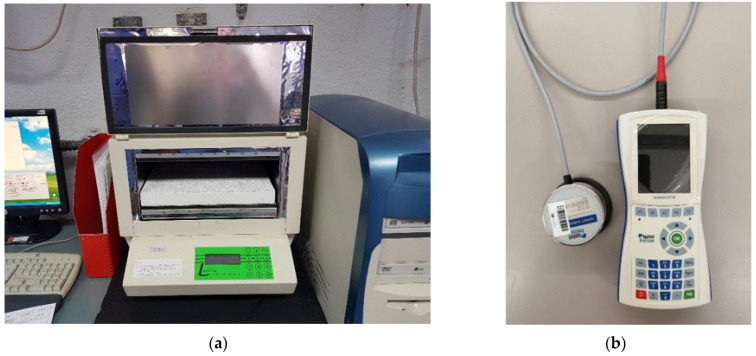
Equipment. (**a**) Heat flow meter (HFM); (**b**) modified transient plane source (MTPS).

The HFM is a steady-state method that consists of placing the specimen between a heated and a cooled plate, establishing a constant temperature gradient. Over a known plate dimension and measuring the rate of heat flow, in compliance with the Seebeck effect, the thermal conductivity of the specimen can be obtained. The equipment that was used was the Fox 304 from LaserComp (Livonia, MI, USA), providing thermal conductivity measurements within the range of 0.005 W/m·K to 0.35 W/m·K, with ±0.2% repeatability and ±0.5% reproducibility (Figure 2a).

The MTPS is based on the release of heat flow impulses into the specimen, followed by the analysis of its thermal response. It can only be applied with the sample in thermal equilibrium with the environment to ensure the temperature response is not affected by the heat flow between the sample and the neighbouring interfaces. This method was applied according to the standard ASTM D7984 [28] and the device ISOMET 2114, from Applied Precision (Bratislava, Slovakia), and a surface probe was used (Figure 2b). For the thermal conductivity, the measuring range of the equipment is 0.04 to 0.3 W/K.m, with an error of 5% of the reading +0.001 W/K.m.

### 2.3. Experimental Framework

Regarding the HFM method, the test was carried out according to the standard UNE EN 12667:2002 [29]. The samples stayed in an oven at 30 °C until mass stabilization (in consecutive 24 h measurements, mass variation inferior to 0.1%). During the measurement, the average laboratory temperature was 22 °C, where the samples were kept for a minimum of 24 h before the experiments.

For the MTPS method, the measurements were carried out in two phases, as follows: (i) phase 1 consisted of assessing the thermal conductivity of the dry material, and (ii) phase 2 consisted of assessing the thermal conductivity of the material under hygroscopic equilibrium with an environment of 80% relative humidity. In each phase, the four sets of samples (reference, PW with a ratio of 50%, PW with a ratio of 60%, and PW with a ratio of 70%) were measured. Before the measurements were carried out, the specimens were dried in an oven at 50 °C, until mass stabilization.

**Figure 3:**
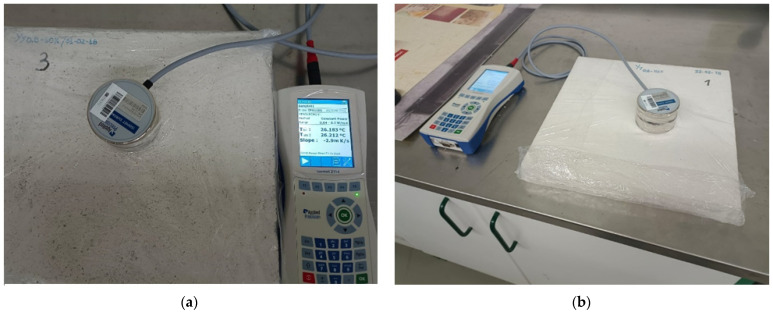
Measurements with the MTPS method. (**a**) Dry material; (**b**) material after 12 days in the climatic chamber at 80% relative humidity.

Phase 1 started immediately after the mass stabilization and after guaranteeing the thermal equilibrium with the environment, with the specimens wrapped in plastic film to avoid adsorption of water vapour from the ambience. For the same reason, the plastic film was kept during the measurements, as illustrated in Figure 3a. For phase 2, the plastic film was removed and the specimens were put inside a climatic chamber at 20 °C and 80% relative humidity for 12 days. After that period, they were weighted, wrapped in plastic film, and their thermal conductivity was assessed (Figure 3b).

In both phases, the laboratory average temperature during the measurements was 23 °C. For each specimen (three for each set, with a total of twelve), three measures of the thermal conductivity were performed, resulting in a total of 36 measurements, with the surface probe always placed in the centre of the sample.

## 3. Results and Discussion

### 3.1. Phase 1—Thermal Conductivity of the Dry Material

**Figure 4:**
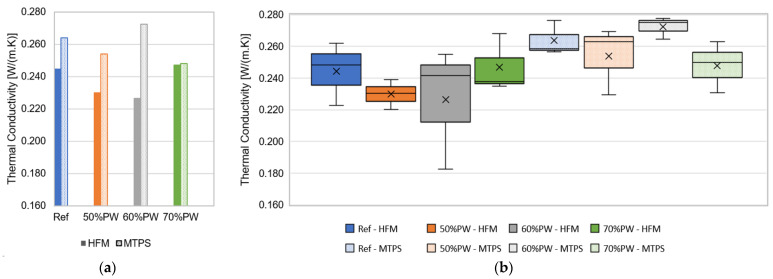
Thermal conductibility for the four sets of gypsum plaster, assessed using the methods HFM (solid colour) and MTPS (patterned colour). (**a**) Box plot representation; (**b**) average values.

The results obtained after applying the heat flow method (HFM) and the modified transient plane source (MTPS) method are presented in Table 1 and Figure 4. The results show that there is some variability in the measurements within each set of samples. Although it can be found for both methods, the values obtained with the MTPS method seem more similar, which may be explained by the smaller area considered for the measurement; in the HFM method, the area that is considered for the calculation of the thermal conductivity is around 900 cm^2^, while in the MTPS method, the area is less than 20 cm^2^. If the mixture of all components of the plaster (gypsum, plastic pellets, and water) did not guarantee a perfectly homogeneous plaster, then the higher the area under study, the greater the variation could be in the values of the thermal conductibility in each measurement for the same set of samples. This issue could potentially be addressed by using a different dosage for the components of the plaster or using additives.

The results also show that, generally, the thermal conductivity values are higher for the MTPS method, although, for the 70%PW set, the difference between the two methods was less significant. The variation tendency of the thermal conductivity with the percentage of incorporated plastic waste differs in the two methods, mainly due to the 60%PW and 70%PW sets. It is possible to observe a relationship between the increase in the percentage of incorporated waste and the decrease in the value of the thermal conductivity, which may be related to the fact that gypsum has a higher thermal conductivity than plastic, as has been stated by other authors [17,18,19,20,21,22]. However, for higher percentages of plastic incorporation, the results become less clear. This may be related to the fact that the mixture of a higher percentage of plastic pellets with gypsum and water is more difficult, implying more heterogeneity of the plaster, affecting the measured values not only for each sample, but also between samples of the same set. The results obtained for the 60%PW set measured using the MTPS method clearly support this statement, as the values are higher than the ones obtained for the reference set. These inconsistencies in the results suggest that more tests need to be carried out, expanding the number of specimens.

### 3.2. Phase 2—Influence of Water Content on Thermal Conductivity

**Figure 5:**
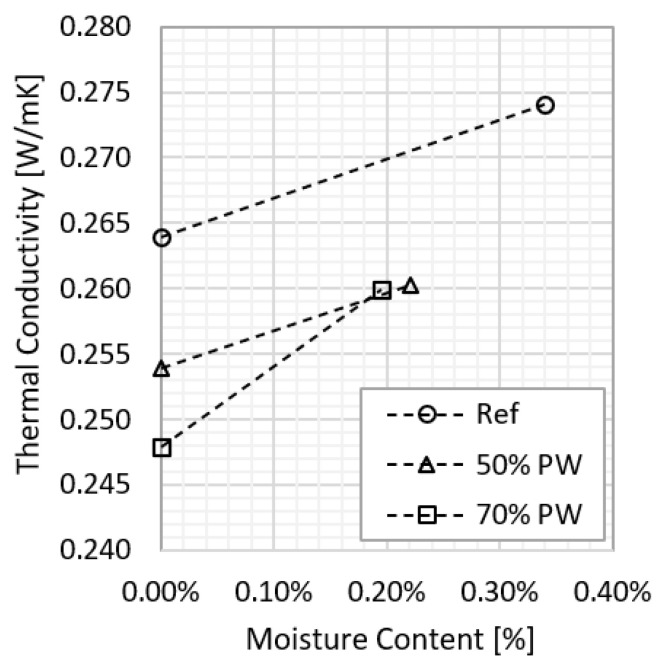
Variation of thermal conductibility with water content for the reference sets, with plastic waste aggregates with a ratio of 50% and with plastic waste aggregates with a ratio of 70%.

The results of phase 2, which consisted of assessing the thermal conductivity using the MTPS method of the four sets of samples, after being in hygrothermal equilibrium with an environment of 80% relative humidity, are shown in Table 2 and in Figure 5. As expected, the results show an increase in the thermal conductivity as the water content rises. However, the 60%PW set does not follow this tendency, which is in line with the deviations found in phase 1. For this reason, this set of samples will be disregarded.

**Figure 6:**
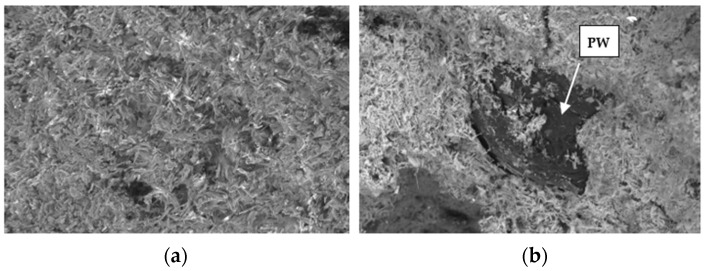
Microscopic images. (**a**) Specimen Ref; (**b**) specimen 50%PW.

**Figure 7:**
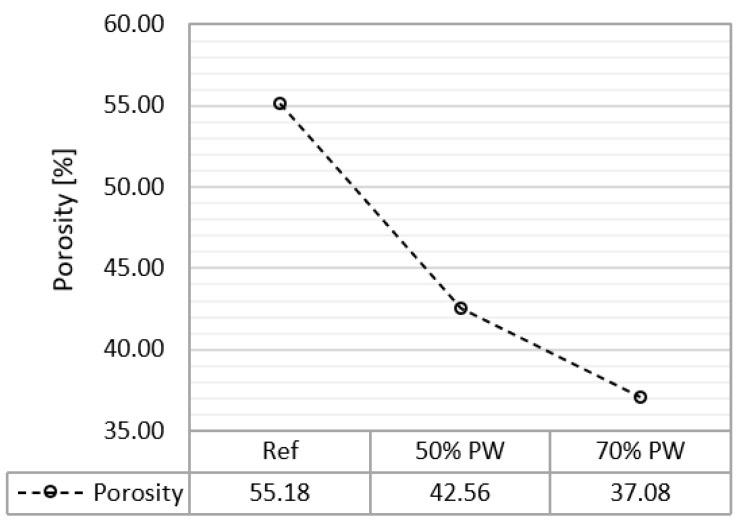
Average porosity for the reference sets, with plastic waste aggregates with a ratio of 50% and with plastic waste aggregates with a ratio of 70%.

Figure 5 shows that the incorporation of plastic waste decreases the water content of the gypsum after being in an environment with 80% relative humidity for 12 days. This is to be expected and is explained by the following two reasons:

- Plastic is a less hygroscopic material than gypsum, so it acts as a barrier to water. In the microscopy image, depicted in Figure 6, the plastic waste can be seen dispersed in the gypsum matrix.
- The smaller number of pores in the gypsum with plastic waste decreases the ability of storing water vapor in the pores. The average results of the mercury porosimetry test, shown in Figure 7, show that the samples with plastic residues are up to 67% less porous than the reference ones.

It can also be observed that the difference in the water content of the two sets of gypsum with plastic residues (50%PW and 70%PW) is not very relevant, which may indicate that this behaviour is independent of the percentage of incorporation, at least, if it is higher than 50%.

Regarding the variation of the thermal conductivity, as previously referred to, it increased with the water content, which is in line with the behaviour of traditional materials (this tendency was observed in both the reference set and in the sets with plastic waste). However, some additional and unexpected conclusions also arise from these results. First, the incorporation of plastic influences the variation of the thermal conductivity with the water content. Second, the values of the thermal conductivity for 80% relative humidity are very similar, regardless of the percentage of plastic waste, which did not occur for the dry material (Table 2). This may be due to the hygric characteristics of the plastic, as it is less hygroscopic.

## 4. Conclusions

After the results of the tests we carried out, it is observed that the thermal properties of the gypsum composites with plastic waste can be affected by the heterogeneity of the composition of the specimens, whereby the greater the amount of pellets, the less homogeneous the mixture. In spite of this heterogeneity, the proposed composites are more efficient, energetically speaking, since their thermal conductivity coefficient is reduced with respect to the reference material by 4–7%, which contributes, in some way, to the energy efficiency of the buildings.

On the other hand, it was found that the method used for the measurement influences the results, reaching values up to 10% higher in the measurement using the transient plane source method with respect to the stationary method. Furthermore, the values obtained with the first method present a lower variability, probably due to the smaller area of contact for the measurement, which may be interesting for assessing heterogeneous materials.

Regarding the thermal performance of the composites based on their moisture content, it has been found that they decrease with a higher saturation, i.e., their thermal conductivity coefficient increases between 2.5 and 4.8%. At the same time, it has been observed that the incorporation of plastic (a non-hygroscopic material) influences the speed and degree of saturation of the specimens, making their thermal conductivity coefficient, with respect to the dry specimen, have a lower variability.

The proposed material, therefore, represents a good opportunity for the manufacture of a more efficient plaster, since it not only minimizes the deposit of plastic waste in landfills, but also because it reduces the amount of water and gypsum stone used in its composition (the more waste, the less water and gypsum) and also contributes as a passive measure in the building envelope and, therefore, in reducing its energy consumption.

## Figures and Tables

**Table 1:** Thermal conductibility, assessed using HFM and MTPS methods, for the four sets of gypsum plaster.

		Thermal Conductibility [W/(m·K)]			
		HFM		MTPS	
Set	Specimen	Meas.	Mean (std)	Meas.	Mean (std)
Ref	S1	0.2620	0.2444 (0.0199)	0.2586	0.2639 (0.0110)
	S2	0.2228		0.2765	
	S3	0.2484		0.2565	
50%PW	S4	0.2389	0.2298 (0.0094)	0.2631	0.2539 (0.0215)
	S5	0.2201		0.2693	
	S6	0.2304		0.2294	
60%PW	S7	0.1827	0.2264 (0.0385)	0.2645	0.2723 (0.0069)
	S8	0.2550		0.2750	
	S9	0.2416		0.2775	
70%PW	S10	0.2379	0.2469 (0.0182)	0.2308	0.2479 (0.0162)
	S11	0.2350		0.2498	
	S12	0.2679		0.2630	

**Table 2:** Thermal conductibility assessed using the MTPS method, and the water content for 80% relative humidity, for the four sets of gypsum plaster.

	DRY	RH = 80%		Variation (%)
	λ [W/(m.K)]	λ [W/(m.K)]	w (%)	
Ref	0.2639	0.2741	0.3391	3.9%
50% PW	0.2539	0.2603	0.2216	2.5%
60% PW	0.2723	0.2623	0.1929	−3.7%
70% PW	0.2479	0.2599	0.1946	4.8%

## Data Availability

The data on which this article is based are supported by different data portals such as national government pages, repositories, and other data sources.
